# Urinary estrogen metabolites and gastric cancer risk among postmenopausal women

**DOI:** 10.1002/cnr2.1574

**Published:** 2021-11-11

**Authors:** M. Constanza Camargo, Minkyo Song, Xia Xu, Isaac Zhao, Joshua N. Sampson, Arash Etemadi, Hermann Brenner, Hwi‐Won Lee, Britton Trabert, Bernd Holleczek, Ben Schöttker, Kathleen Spaid, Sanford M. Dawsey, Sangjun Lee, Takaya Shimura, Sue K. Park, Reza Malekzadeh, Daehee Kang, Charles S. Rabkin

**Affiliations:** ^1^ Division of Cancer Epidemiology and Genetics National Cancer Institute Rockville Maryland USA; ^2^ Cancer Research Technology Program, Leidos Biomedical Research, Inc. Frederick National Laboratory for Cancer Research Frederick Maryland USA; ^3^ Division of Preventive Oncology German Cancer Research Center and National Center for Tumor Diseases Heidelberg Germany; ^4^ Division of Clinical Epidemiology and Ageing Research German Cancer Research Center Heidelberg Germany; ^5^ Department of Preventive Medicine Seoul National University College of Medicine Seoul South Korea; ^6^ Department of Laboratory Medicine, Clinical Center National Institutes of Health Bethesda Maryland USA; ^7^ Department of Gastroenterology and Metabolism Nagoya City University Graduate School of Medical Sciences Nagoya Japan; ^8^ Digestive Disease Research Center, Digestive Diseases Research Institute Tehran University of Medical Sciences Tehran Iran

**Keywords:** estradiol, estrogens, estrone, gastric cancer, sex hormones

## Abstract

**Background:**

The overall incidence of gastric cancer in women is half that in men for most global populations. Sex hormone pathways may be involved in carcinogenesis and estrogens have been postulated to protect women against gastric cancer.

**Aim:**

To evaluate associations of gastric cancer with estrogen metabolites in postmenopausal women.

**Methods and results:**

We performed an analysis of 233 gastric cancer cases and 281 age‐matched controls from three prospective cohorts and two case‐control studies of early‐stage gastric cancer, mainly conducted in high‐risk Asian populations. Fifteen estrogen‐parent (estrone and estradiol) and ‐metabolite analytes (2‐hydroxyestrone, 2‐hydroxyestradiol, 2‐hydroxyestrone‐3‐methyl ether, 4‐hydroxyestrone; 4‐methoxyestrone, 4‐methoxyestradiol, 2‐methoxyestrone, 2‐methoxyestradiol, estriol, 16α‐hydroxyestrone, 16‐ketoestradiol, 16‐epiestriol, and 17‐epiestriol) were measured in spot urines using liquid chromatography–tandem mass spectrometry. Odds ratios for association with each marker were estimated by logistic regression. Heterogeneity was assessed by Cochran's Q test. Study‐specific odds ratios were pooled by fixed‐effects meta‐analysis. Urinary levels of estrogen‐related molecules were not associated with gastric cancer (adjusted odds ratios ranged from 0.87 to 1.27; *p*‐values >.05), with low between‐study heterogeneity (*p*‐values >.1) for all but two metabolites (2‐hydroxyestrone‐3‐methyl ether and 2‐methoxyestradiol).

**Conclusion:**

To date, this is the first comprehensive assessment of endogenous estrogens with gastric cancer risk in women. Estrogens do not appear to have an etiologic role in gastric cancer risk among postmenopausal women. Given the complex network of sex steroid hormones and their extreme variation over the lifespan, further evaluation of this hypothesis is warranted.

## 1 INTRODUCTION

Gastric cancer represents the fourth leading cause of cancer death worldwide.^1^ The overall incidence of gastric cancer in women is half that in men for most populations. This sex difference cannot be fully explained by variations in *Helicobacter pylori* infection, sociodemographic or environmental factors.^2^

Sex steroid hormone pathways may be involved in carcinogenesis. Our previous meta‐analysis of menstrual and reproductive factors identified decreased gastric cancer risk among women with a longer time between menarche and menopause or exposure to hormone therapy.^3^ These surrogate measures suggest extended exposure to estrogens may protect against gastric cancer. We directly tested this hypothesis in a multicenter case‐control study by evaluating the associations between conjugated estrogens and estrogen metabolites excreted in urine and gastric cancer risk in postmenopausal women. Total concentrations of individual estrogen moieties are moderately correlated between urine and serum in postmenopausal women.^4^

## 2 METHODS

### 2.1 Study population

Our analysis included 216 women from three population‐based prospective studies (Korean Multicenter Cancer Cohort,^5^ Iranian Golestan Cohort,^6^ and German ESTHER Cohort^7^) and 298 women from two case–control studies of early‐stage cancer (American Joint Committee on Cancer clinical stages 1A or 1B) from Japan^8^ and Korea. Gastric cancer cases were identified based on the International Classification of Diseases (10th Revision), codes C16. In total, 233 postmenopausal women (with known postmenopausal status or age 60 years or older) with gastric cancer (85% non‐cardia tumors) were frequency‐matched by study and 5‐year age category to 281 gastric cancer‐free controls. For the prospective samples, the median time from urine collection to cancer diagnosis ranged from 5 (Golestan cohort) to 7.4 (ESTHER cohort) years. This analysis was restricted to women not taking exogenous hormones and included all available gastric cancer cases (at least five per study) at the time of selection. Spot urine specimens were collected at enrollment in prospective studies and pre‐treatment in case‐control studies.

### 2.2 Laboratory assays

Urinary levels of parent estrogens (estrone and estradiol) and 13 metabolites (2‐hydroxyestrone, 2‐hydroxyestradiol, 2‐hydroxyestrone‐3‐methyl ether, 4‐hydroxyestrone; 4‐methoxyestrone, 4‐methoxyestradiol, 2‐methoxyestrone, 2‐methoxyestradiol, estriol, 16α‐hydroxyestrone, 16‐ketoestradiol, 16‐epiestriol, and 17‐epiestriol) were simultaneously measured in urine (500 μL aliquots) using liquid chromatography–tandem mass spectrometry assay,^4^ the gold standard method for hormone assessment. Estrogen concentrations were normalized to creatinine levels. Laboratory personnel were blinded to the case‐control status. A quality control set of 20 masked duplicate samples and four laboratory replicates (2 pre‐menopausal, 1 post‐menopausal, 1 male) totaling 60 samples was tested along with the study samples. Intraclass correlation coefficients (ICC) for log‐transformed metabolite concentrations were calculated using a linear mixed model. Within‐batch ICCs were ≥79% for 13 of the 15 analytes (62% for 4‐methoxyestradiol and 56% for 2‐methoxyestrone).

### 2.3 Statistical analysis

We calculated pairwise Spearman's rank correlation coefficients among urinary metabolites using the controls. We used logistic regression to evaluate the association between the risk of gastric cancer and the log_2_ concentration of each metabolite. We report the study‐specific odds ratios (OR) and 95% confidence intervals from models that stratify by batch and adjust for age (continuous), body mass index (continuous) and, when available, smoking (ever vs. never), alcohol use (ever vs. never), education (none vs. any) and family history of gastric cancer (yes vs. no). This information was collected at enrollment in prospective studies and pre‐treatment in case–control studies. Study‐specific ORs were averaged via fixed‐effects meta‐analysis using the “metafor” R package, with heterogeneity assessed by Cochran's Q test. A *p*‐value less than .05 was considered statistically significant, except for test of heterogeneity for which we used a *p*‐value cut‐off of .1. Given the exploratory nature of our study, results were not corrected for multiple comparisons.

## 3 RESULTS

Baseline characteristics of gastric cancer cases and controls are presented in Table 1. Most cases represented noncardia gastric cancer.

**TABLE 1:** Demographic and selected characteristics of gastric cancer cases and controls in three cohort and two case‐control studies

Characteristics	Cohort studies						Early‐stage case‐control studies				Overall	
	Iran		Korea		Germany		Korea		Japan			
	Cases *n* = 38	Controls *n* = 78	Cases *n* = 29	Controls *n* = 52	Cases *n* = 9	Controls *n* = 10	Cases *n* = 142	Controls *n* = 128	Cases *n* = 15	Controls *n* = 13	Cases *n* = 233	Controls *n* = 281
Age in years, mean (SD)	63.4 (7.4)	62.5 (8.1)	63.8 (6.3)	61.3 (6.4)	64.6 (5.0)	65.2 (5.2)	64.6 (8.0)	57.2 (5.9)	72.1 (6.1)	71.8 (6.1)	64.8 (7.7)	60.5 (7.5)
BMI, mean (SD)	27.5 (5.8)	27.5 (5.4)	24.5 (3.0)	24.6 (3.6)	29.5 (5.7)	29.4 (4.6)	23.9 (3.2)	25.0 (3.3)	22.9 (3.4)	21.2 (2.6)	24.8 (4.2)	25.6 (4.4)
Ever smoked, *n* (%)	2 (5)	2 (3)	3 (10)	3 (6)	0 (0)	2 (20)	6 (4)	6 (5)	2 (13)	1 (8)	13 (6)	14 (5)
Ever drank alcohol, *n* (%)	0 (0)	0 (0)	6 (21)	11 (21)	2 (22)	4 (50)	29 (20)	45 (36)	2 (13)	2 (15)	39 (17)	62 (22)
Any educational degree, *n* (%)	0 (0)	10 (13)	19 (66)	33 (63)	9 (100)	10 (100)	126 (89)	114 (91)	–	–	154 (71)	167 (62)
Relative with gastric cancer, *n* (%)	1 (3)	1 (1)	–	–	0 (0)	1 (10)	27 (20)	14 (12)	1 (7)	4 (31)	29 (14)	20 (9)
Median time from urine collection to cancer diagnosis, years	5	–	5.9	–	7.4	–	–	–	–	–	–	–
Noncardia localization, *n* (%)	16 (43)^a^	–	–	–	8 (89)	–	135 (95)^b^	–	13(87)	–	172 (85)	–

Abbreviations: BMI, body mass index; SD, standard deviation.

^a^ 1 unspecified tumor is not included.

^b^ Tumors localized to the fundus are not included.

The percentage of urine samples with missing measurements (due to misalignment of co‐eluting peaks and below lower limit of quantitation) ranged between 4% (2‐methoxyestradiol) and 34% (4‐hydroxyestrone), with a median of 5%. Most estrogen markers had a statistically significant correlation with each other in controls (median correlation coefficient = 0.45; interquartile range = 0.36–0.56).

**FIGURE 1:**
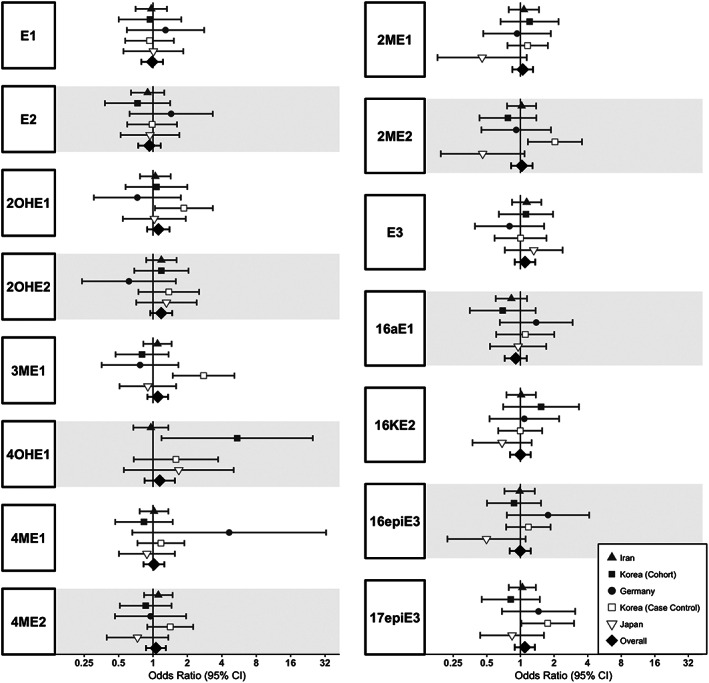
Forest plots of overall and study‐specific associations between urinary estrogens/estrogen metabolites and gastric cancer among postmenopausal women, combining cohort (solid symbols) and early‐stage case‐control (open symbols) studies. Odds ratios (ORs) and 95% confidence intervals (CIs) for gastric cancer per log2 concentration increase of each estrogen were estimated from logistic regression models adjusted for age, body mass index, smoking habits, alcohol use, and family history of gastric cancer, as available (Table 1). Abbreviations: E1, estrone; E2, estradiol; 2OHE1, 2‐hydroxyestrone; 2OHE2, 2‐hydroxyestradiol; 3ME1, 2‐hydroxyestrone‐3‐methyl ether; 4OHE1, 4‐hydroxyestrone; 4ME1, 4‐methoxyestrone; 4ME2, 4‐methoxyestradiol; 2ME1, 2‐methoxyestrone; 2ME2, 2‐methoxyestradiol; E3, estriol; 16aE1, 16α‐hydroxyestrone; 16KE2, 16‐ketoestradiol; 16epiE3, 16‐epiestriol; 17epiE3, 17‐epiestriol. *P*_Heterogeneity_ < .10 for 3ME1 and 2ME2

Figure 1 shows the 15 estrogen markers and their associations with gastric cancer risk. Adjusted ORs from the meta‐analyses ranged from 0.87 to 1.27 but were not statistically significant. There was low between‐study heterogeneity (*p*‐values >.1) for all but two markers, 2‐hydroxyestrone‐3‐methyl ether (*p* = .03) and 2‐methoxyestradiol (*p* = .04).

## 4 DISCUSSION

The reasons for sex differences in gastric cancer are not well elucidated. Despite the plausible biologic evidence from epidemiologic,^3^ laboratory and clinical studies,^9^, ^10^ our multicenter study found that urinary concentrations of parent estrogens and their metabolites in postmenopausal women were not associated with gastric cancer risk. We found a similar null association with estradiol in a case‐control study of invasive noncardia gastric cancer using diagnostic blood samples.^11^

This study is the first epidemiologic analysis that directly evaluated estrogen metabolism in gastric carcinogenesis in women using samples collected prior to cancer diagnosis or asymptomatic localized disease (with limited opportunity for reverse causality). We also used a sensitive and specific mass spectrometry method to accurately quantitate estrogens. Our study is informative as it mainly included women from high gastric cancer risk populations in East Asia. Despite these strengths, our study was limited by small numbers for subgroup analyses.

In conclusion, our study did not find evidence of associations between postmenopausal estrogen levels and gastric cancer risk. Future studies should consider estrogen effects through the lifespan as well as associations with gastric preneoplastic conditions, particularly intestinal metaplasia. Notably, premenopausal estrogen levels are highly dynamic, thus studies of their associations are extremely challenging. A better understanding of sex hormone effects on gastric cancer may reveal common mechanisms of carcinogenesis responsible for other estrogen‐related malignancies.

## CONFLICT OF INTEREST

The authors have stated explicitly that there are no conflicts of interest in connection with this article.

## AUTHOR CONTRIBUTIONS

Concept and design, M.C.C.,M.S., and C.S.R.; Acquisition, analysis, or interpretation of data, X.X., A.E., H.B., H.‐W.L., B.T., B.H., B.S., K.S., S.M.D., S.L., T.S., S.K.P., R.M., and D.K.; Drafting of the manuscript, M.C.C., M.S., I.Z., and C.S.R.; Critical revision of the manuscript for important intellectual content: All authors; Statistical analysis, I.Z., and J.N.S.; Supervision, C.S.R.

## ETHICAL STATEMENT

All individuals provided informed consent. The original studies were approved by the corresponding Institutional Review Boards. The present case‐control analysis was exempted from institutional review board evaluation by the U.S. National Institutes of.

Health Office of Human Subjects Research.

## Data Availability

Data in this study are available on request from the authors.
